# Study protocol: infectious diseases consortium (I3D) for study on integrated and innovative approaches for management of respiratory infections: respiratory infections research and outcome study (RESPIRO)

**DOI:** 10.1186/s12879-023-08795-8

**Published:** 2024-01-23

**Authors:** Dorothy Hui Lin Ng, Travis Ren Teen Chia, Barnaby Edward Young, Sapna Sadarangani, Ser Hon Puah, Jenny Guek Hong Low, Gabriel Zherong Yan, Yin Mo, Nicholas Jinghao Ngiam, Samuel Sherng Young Wang, Yan Tong Loo, Faith Evangeline Jie Qi Ong, Andrew Yunkai Li, Sharlene Ho, Lisa Ng, Paul Anantharajah Tambyah, Tsin Wen Yeo

**Affiliations:** 1 Department of Infectious Diseases, Singapore General Hospital, Singapore, Singapore; 2 National Centre of Infectious Diseases, Tan Tock Seng Hospital, Singapore, Singapore; 3 Department of Infectious Diseases, Tan Tock Seng Hospital, Singapore, Singapore; 4 Lee Kong Chian School of Medicine, Nanyang Technological University, Singapore, Singapore; 5 Department of Respiratory & Critical Care Medicine, Tan Tock Seng Hospital, Singapore, Singapore; 6 Viral Research and Experimental Medicine Centre (ViREMiCS), SingHealth Duke-NUS Academic Medical Centre, Singapore, Singapore; 7 Programme in Emerging Infectious Diseases, Duke-NUS Medical School, College Road, Singapore, Singapore; 8 Division of Infectious Diseases, National University Hospital, Singapore, Singapore; 9 Division of Respiratory & Critical Care Medicine, Department of Medicine, National University Hospital, Singapore, Singapore; 10 Singapore Immunology Network, Agency for Science, Technology and Research, Singapore, Singapore; 11 Infectious Diseases Translational Research Programme, Department of Medicine, Yong Loo Lin School of Medicine, National University of Singapore, Singapore, Singapore

**Keywords:** Study protocol, Community-acquired pneumonia, Epidemiology, Pathogenesis

## Abstract

**Background:**

Community-acquired respiratory infections are a leading cause of illness and death globally. The aetiologies of community-acquired pneumonia remain poorly defined. The RESPIRO study is an ongoing prospective observational cohort study aimed at developing pragmatic logistical and analytic platforms to accurately identify the causes of moderate-to-severe community-acquired pneumonia in adults and understand the factors influencing disease caused by individual pathogens. The study is currently underway in Singapore and has plans for expansion into the broader region.

**Methods:**

RESPIRO is being conducted at three major tertiary hospitals in Singapore. Adults hospitalised with acute community-acquired pneumonia or lower respiratory tract infections, based on established clinical, laboratory and radiological criteria, will be recruited. Over the course of the illness, clinical data and biological samples will be collected longitudinally and stored in a biorepository for future analysis.

**Discussion:**

The RESPIRO study is designed to be hypothesis generating, complementary to and easily integrated with other research projects and clinical trials. The detailed clinical database and biorepository will yield insights into the epidemiology and outcomes of community-acquired lower respiratory tract infections in Singapore and the surrounding region and offers the opportunity to deeply characterise the microbiology and immunopathology of community-acquired pneumonia.

**Supplementary Information:**

The online version contains supplementary material available at 10.1186/s12879-023-08795-8.

## Background

Community-acquired respiratory infections represent a significant global health challenge, contributing significantly to illness and mortality worldwide. Seasonal respiratory infections continue to afflict the human population, and the lack of universally effective long-term vaccines or preventative measures exacerbates the problem. Moreover, new viral respiratory pathogens are emerging with increasing frequency. These can have devastating impacts, as underscored by the epidemics of Severe Acute Respiratory Syndrome Coronavirus (SARS-CoV), the Middle East Respiratory Syndrome Coronavirus (MERS-CoV) and the COVID-19 pandemic caused by SARS-CoV-2 [1, 2]. In both Singapore and the United States, pneumonia stands out as a primary infectious cause of hospitalisation and death among adults [3, 4]. The annual incidence of community-acquired pneumonia (CAP) requiring hospitalisation stands at approximately 25 cases per 10,000 adults, incurring annual medical costs surpassing US$10 billion [4, 5]. This healthcare burden is mirrored in many other countries.

Despite the widespread impact of these diseases, significant gaps persist in our understanding of the array of pathogens responsible for community-acquired respiratory infections. Over half of community-acquired lower respiratory tract infections lack identified microbial causes [6, 7]. This may be attributed to factors such as limited diagnostic samples or testing, insensitive diagnostic tests, antibiotic use before specimen collection, non-infectious conditions mimicking respiratory infections, and unidentified or uncharacterised pathogens. Consequently, most patients are treated empirically, which carries the risk of antibiotic overuse and the development of antibiotic resistance. Furthermore, within this group of unknown pathogens lies the potential threat of novel pathogens with epidemic or pandemic potential, without an optimised system for surveillance or early identification, particularly for moderate to severe cases. Most sentinel surveillance systems tend to focus on either fatal cases or mild, community-based cases with primarily upper respiratory symptoms [8]. Additionally, many of the known pathogens’ early and long-term effects remain poorly understood due to the lack of robust epidemiological data.

This study aims to address these knowledge gaps by pursuing the following objectives: (1) To evaluate the causes and outcomes of moderate-to-severe CAP in Singapore and the surrounding region; (2) To establish a repository of biological samples to validate novel diagnostics; (3) To gain a mechanistic understanding of host-pathogen disease processes in moderate-to-severe CAP. By combining pathogen information with host disease phenotypes, this approach will enable the development of a “precision medicine” approach to pathogen discovery and the potential identification of novel adjunctive therapies.

## Methods/Design

RESPIRO is a multi-centre, observational, prospective study. The protocol is intentionally structured to be adaptable for implementation in various hospital settings. It accommodates a range of study intensities to facilitate the swift collection and analysis of clinical data and biological samples. In Singapore, participants will be primarily recruited from three major tertiary hospitals: National Centre for Infectious Diseases/Tan Tock Seng Hospital, National University Hospital, and Singapore General Hospital. Recruitment from these sites began in June 2022 and is ongoing. We are also in the process of planning additional study sites in and beyond Singapore. Recognising that each site may differ in its infrastructure, resources, and capacity, allowances are made for study sites to adjust the intensity of data and sample collection according to their unique capabilities. The approach to data analyses will be aligned with available data and samples at each site. This study is designed to have flexible and seamless integration with other ongoing research projects and clinical trials.

## Study population

This study focuses on individuals with moderate-to-severe CAP, excluding cases of healthcare-associated pneumonia and pulmonary infections in severely immunocompromised hosts.

Eligibility criteria for patient inclusion are as follows:

1. Evidence of acute infection within 7 days (defined as self-reported fever/chills, documented fever (≥ 38^o^C) or hypothermia (< 35.5^o^C), leucocytosis (WBC > 11,000/mm^3^) or leucopenia (WBC < 3000/mm^3^), or new altered mental status).
2. Evidence of acute respiratory illness (defined as new cough or sputum production, dyspnoea, tachypnoea (> 25 breaths/minute), abnormal lung examination, respiratory failure).
3. Evidence of pneumonia on lung imaging (chest radiography or computed tomography) within 48 h before or after admission.
4. Age ≥ 21 years old.
5. Patient or legal representative is able to provide informed consent.

Patients will be ineligible for the study if any of the following criteria apply:

1. Recent hospitalisation (defined as < 28 days for immunocompetent patients and < 90 days for immunosuppressed patients).
2. Functionally dependent nursing home residents.
3. Clear alternative diagnosis in the opinion of the treating physician and/or study team.
4. Patients with tracheostomy-in-situ.
5. Patients with cystic fibrosis.
6. Neutropenia due to cytotoxic chemotherapy.
7. Solid organ transplant or haematopoietic stem-cell transplant in the previous 90 days.
8. Active graft-versus-host disease or bronchiolitis obliterans.
9. Human immunodeficiency virus infection with CD4 count < 200/mm^3^.
10.  Current COVID-19 infection confirmed by SARS-CoV-2 PCR.
11.  Recent enrolment in the current study within 28 days.

Enrolled patients may be terminated by the study team for the following reasons:

1. Illness is subsequently confirmed to be the result of infection with a pathogen that is not relevant to the study objectives, and who have no indication or likelihood of co-infection with a relevant pathogen.
2. Any new information becomes available that makes further participation unsafe.
3. Termination of the study.

## Data and sample collection

Clinical, laboratory and radiological data will be collected directly from the hospitals’ medical records of the participating hospitals. Patient symptoms will be assessed using structured patient surveys and questionnaires. Serial biological samples will be collected according to the schedule outlined in Table 1. The schedule includes a range of 1 to 13 sample sets, accommodating variations in the capacity of each study site.

**Table 1:** Summary of study schedule

Study activity	Screening	Inpatient follow-up^a^		Samples, symptoms questionnaire, and quality-of-life survey			Final visit
	D1 (priority)	Every other day (D3-15) ± 1 day	D21 ± 7 days	D28 ± 14 days (priority)	D90 ± 30 days (priority)	D180 ± 60 days	D360 ± 90 days
Eligibility assessment	X						
Informed consent	X						
Demographics	X						
Medical history	X						
Clinical data collection	X	Daily until end of hospital stay		X	X	X	X
Symptoms questionnaire	X	Every other day until end of hospital stay. (Day 1 to 7 of study will be prioritised.)		X	X	X	X
Quality-of-life survey				X	X	X	X
Sample collection^b^							
Blood^c^	X	X	X	X	X	X	X
Respiratory samples	X	X	X	X	X	X	X
Urine^c^	X	X	X	X	X	X	X
Stool^c^	X	X	X	X	X	X	X
Rarely other biological samples (e.g. tears)	X	X	X	X	X	X	X
Residual samples^d^	X						

^a^Serial sampling will stop when the patient’s acute illness resolves, or when they are discharged from the hospital: next samples taken will be at 28, 90, 180, 360 days post-recruitment

^b^In the event of resource limitations requiring sampling frequency to decrease or for any other reasons (e.g. the patient’s personal preference to have minimal sampling for research purposes), samples will be prioritised at recruitment, day 28, and day 90

^c^Up to a maximum of 30 mL (approximately 6 teaspoons) of blood may be drawn for each time point as well as 10 mL of urine and 10 g of stool (approximately 2 teaspoons)

^d^Residual samples are leftover samples from routine clinical testing. The study team will coordinate with the clinical laboratory team to keep any residual samples that may be useful to this study

All data will be anonymised at the hospital site. Each patient will be assigned a unique study number, and the unique study number and anonymised data will be submitted electronically to a Research Electronic Data Capture (REDCap) electronic case report form (eCRF) by the hospital study staff. Additionally, an enrolment log, containing the patient’s name, date of birth, hospital identification number, and unique study number, will be maintained separately.

Samples will be processed and stored for use in subsequent studies related to pathogen research, host immune responses, and biomarker discovery.

## Study visits and procedures

### Acute Illness and index admission

Clinical, laboratory and radiological data will be extracted from the hospitals’ medical records. Patient symptoms will be assessed using structured patient surveys and questionnaires on day 1 (the day of recruitment), and subsequently every other day until day 7 of enrolment or the patient’s discharge from the hospital, whichever occurs first.

Serial samples, including blood, sputum, urine, and nasopharyngeal/oropharyngeal swabs, will be collected on day 1 and at various intervals throughout the index admission, day 28 and during convalescence. Wherever feasible, additional samples, such as nasopharyngeal aspirates, flocked nose and throat swabs, samples from infected sites (like inflamed oropharynx or conjunctiva), and any residual pathogen samples collected for clinical care, will also be obtained. If bronchoscopy with bronchoalveolar lavage is conducted as part of clinical care and with patient consent, extra samples for research purposes will be obtained. Endotracheal aspirates will be obtained from intubated patients.

Resolution of acute illness will be defined as the clearance of the pathogen from appropriate samples, return of systemic inflammatory response markers to normal range values, and one of the following criteria: (1) recovery from organ failure(s)/need for organ support, (2) resolution of the presenting complaint(s), (3) a return to the patient’s pre-illness lifestyle, or upon the patient’s discharge from the hospital.

### Follow-up visits

Patients will be scheduled for follow-up visits on days 28, 90, 180, and 360. Clinical, laboratory and radiological data collection, assessment of patient symptoms, and sample collection will be performed as needed on those visits.

The frequency and extent of follow-up visits and procedures will depend on the availability of resources and capacity at each hospital site. Hospital sites that may face limitations can adjust the frequency of study visits or procedures. In such cases, the collection of samples will be prioritized for day 28 and 90 post-recruitment.

## Outcome variables

### Primary outcomes

1. To determine the frequency, seasonal patterns, and burden of disease due to different respiratory pathogens causing acute CAP in Singapore including unknown pathogens.
2. To determine the factors contributing to severity of illness, length of stay, mortality, and secondary infections.

### Secondary outcomes

1. To describe the following for each individual pathogen:
  - Clinical features and risk factors for severe disease.
  - Response to treatment including supportive care and novel therapeutics.
  - Pathogen replication, excretion and evolution, and pathogen determinants of severity and transmissibility.
  - Host responses to infection and therapy, and host factors associated with progression or severity.

## Power/sample size

This study, aiming to provide a comprehensive description of the epidemiology and outcomes of acute community-acquired respiratory tract infections, will strive to enrol as many patients as possible without predetermined limits. Based on our clinical experience and the current available funding of the study until 2026, we anticipate recruiting up to 500 subjects from each of the three participating Singapore study sites throughout the study’s duration. As the existing grant approaches expiration, our study team may actively seek new sources of funding to ensure the study’s continuation.

## Statistical analysis

Enrolled patients will be categorised based on the aetiology and severity of their disease. Patients with unconfirmed aetiologies will be analysed as a separate group. Standard statistical methods appropriate to the data distribution and types will be employed to compare differences between these groups.

## Protection of human subjects and ethical approval

Patients who meet the study’s inclusion and exclusion criteria will be invited to participate. Informed consent will be sought from the patients themselves or their legal representative. The informed consent form will be provided in English. For participants who are unable to read or understand English, verbal translation of the document and discussion will occur in the presence of an impartial witness. A concise informed consent form in Chinese or Malay will also be utilized. Our study staff will clearly communicate all aspects of the study, including procedures, potential risks, benefits, the right to withdraw, and alternative options, allowing patients or their legal representatives ample time for discussion and questions. For patients whose capacity to consent may change during the study, informed consent will be revisited when they are able to make independent decisions. The consent process will adhere to the principles of Good Clinical Practice (GCP) and align with the regulations governing clinical research at the recruiting hospital sites. Prospective patients are under no obligation to participate and can decline or withdraw without facing any negative implications. All patients will receive routine clinical care, regardless of their participation in the study. This study is approved by the National Healthcare Group Domain Specific Review Board (NHG DSRB Ref: 2021/00970). The study protocol was peer-reviewed as part of the funding process. This study adheres to the principles outlined in the Helsinki Declaration of 1964, along with its subsequent amendments.

## Discussion

CAP remains a pressing global concern, with substantial morbidity and mortality, while the potential emergence of novel pathogens emphasizes the need for better diagnostics and research capabilities. If the aetiology is discovered for the approximately half of cases of CAP without a known cause, it could potentially open new avenues for diagnostics and therapeutics for both known and unknown pathogens. This is vital not only for developing precise diagnostic tools, prognostic biomarkers and therapeutic strategies, but also for unravelling the immediate and long-term consequences of specific infections [9, 10]. In this context, RESPIRO aims to become a proactive and collaborative logistical and analytic platform focused on systematically identifying the infectious agents responsible for CAP in adults in Singapore and the surrounding region, whether known or unknown. Detailing the aetiological agents is critical for assigning appropriate therapy for the identified pathogen, potentially reducing unnecessary antibiotic use, a major driver of antimicrobial resistance in the region. The wealth of longitudinal clinical data and biological samples gathered will also provide an opportunity to deeply characterize the host immune response in relation to pathogen, disease severity, and clinical outcomes.

This is the first nationwide, prospective effort to characterize the epidemiology of moderate-to-severe acute lower respiratory tract infections for adults in Singapore. Beyond its main objectives, the integrated approach and network capabilities will be essential for informing and improving critical aspects of clinical management interventions. For instance, the identification of biomarkers may help to streamline the triage and management decisions of hospitalised patients with acute CAP. As exemplified by the UK Randomised Evaluation of COVid-19 thERapY (RECOVERY) trial during the COVID-19 pandemic, where dexamethasone had varying effects in different patient groups [11], the need for targeted patient selection in prescribing treatments is evident. In the event of a respiratory infectious outbreak or epidemic, we can leverage on the ongoing RESPIRO platform for real-time immunoprofiling of the patients at different stages, pinpointing distinct patient groups that could benefit from specific interventions. Moreover, the establishment of a nationwide network will enable the identification of early signals indicating the spread of clonal respiratory infections across time and space. This capability will allow us to assess infectivity risk and implement appropriate infection control measures, thereby swiftly curbing its spread.

Due to its design, which intentionally accommodates variance to maximise participation across various study sites, we anticipate a considerable amount of missing data. This can reduce the overall statistical power of the study and can lead to biased estimates, potentially resulting in invalid conclusions. To address this, numerous procedures will be implemented to maximise data quality and protocol standardisation. These procedures include prioritizing specific time-points in resource-limited settings, defining data collected in the REDCap eCRF with a detailed data dictionary, direct recording of survey and questionnaire data into the REDCap eCRF, integrating quality control checks for critical data points, eliminating paper CRFs, and conducting random monitoring of study sites and data. Statistical analyses will be carried out to minimize assumptions about missing data.

The evidence generated by RESPIRO is intended to inform the development of clinical guidelines and recommendations for policymakers, extending beyond routine CAP treatment guidelines to include pathogen-specific protocols. Data will be periodically shared through various channels, including workshops, abstracts, presentations, and publications. Beyond this, RESPIRO will establish a robust network for respiratory disease surveillance, pave the way for the advancement of innovative diagnostics, therapeutics, and models to understand host-pathogen interactions, and provide a platform for conducting pre-clinical and clinical trials at both national and regional levels.

### Electronic supplementary material

Below is the link to the electronic supplementary material.

Supplementary Material 1

## Data Availability

This is a study protocol manuscript. Not appliable.

## List of abbreviations

RESPIRO: Respiratory Infections Research and Outcome study
CAP: Community-acquired pneumonia
eCRF: Electronic case report form
SARS-CoV: Severe Acute Respiratory Syndrome Coronavirus
MERS-CoV: Middle East Respiratory Syndrome Coronavirus
COVID-19: Coronavirus disease of 2019
REDCap: Research Electronic Data Capture
eCRF: Electronic case report form
WBC: White blood cell
CD4: Cluster of differentiation 4
